# UGT1A1 and UGT1A9 Are Responsible for Phase II Metabolism of Tectorigenin and Irigenin In Vitro

**DOI:** 10.3390/molecules27134104

**Published:** 2022-06-26

**Authors:** Ji Li, Zhangyao Xu, Jifeng Gu

**Affiliations:** 1 Department of Radiation Oncology, Eye and ENT Hospital, Fudan University, Shanghai 200031, China; ji.li@fdeent.org; 2 Department of Pharmacy, Eye and ENT Hospital, Fudan University, Shanghai 200031, China; zhangyao.xu@fdeent.org; 3 Shanghai Key Laboratory of Bioactive Small Molecules, School of Basic Medical Sciences, Fudan University, Shanghai 200031, China

**Keywords:** tectorigenin, irigenin, glucuronidation, human liver microsomes, response factor method

## Abstract

Tectorigenin and irigenin are biologically active isoflavones of *Belamcanda chinensis* (L.) DC. Previous studies indicated that both compounds could be metabolized in vivo; however, the kinetic parameters of enzymes involved in the metabolization of tectorigenin and irigenin have not been identified. The aim of this study was to investigate UGTs involved in the glucuronidation of tectorigenin and irigenin and determine enzyme kinetic parameters using pooled human liver microsomes (HLMs) and recombinant UGTs. Glucuronides of tectorigenin and irigenin were identified using high-performance liquid chromatography (HPLC) coupled with mass spectrometry and quantified by HPLC using a response factor method. The results showed that tectorigenin and irigenin were modified by glucuronidation in HLMs. One metabolite of tectorigenin (M) and two metabolites of irigenin (M1 and M2) were detected. Chemical inhibition and recombinant enzyme experiments revealed that several enzymes could catalyze tectorigenin and irigenin glucuronidation. Among them, UGT1A1 and UGT1A9 were the primary enzymes for both tectorigenin and irigenin; however, the former mostly produced irigenin glucuronide M1, while the latter mostly produced irigenin glucuronide M2. These findings suggest that UGT1A1 and UGT1A9 were the primary isoforms metabolizing tectorigenin and irigenin in HLMs, which could be involved in drug–drug interactions and, therefore, should be monitored in clinical practice.

## 1. Introduction

*Belamcanda chinensis* (L.) DC. is a perennial herbaceous plant [1]. In 2005, *Belamcanda chinensis*, the sole species in the genus *Belamcanda* [2], was transferred to the genus *Iris* and renamed *Iris domestica.* Its Chinese name is Shegan, and it is widely used in China and other East Asian countries [3] because of its heat-clearing and detoxifying effects, relieving sore throat and resolving mucus accumulation. It is often used as a principal ingredient in combination with other traditional Chinese medicines for the treatment of respiratory diseases in clinics. Modern pharmacological studies indicate that Shegan has a variety of pharmacological activities, including anti-inflammatory, antioxidant, free-radical scavenging, anticancer, antibacterial, and estrogenic effects [2]. Recently, Shegan was included in the European Pharmacopeia [3].

**Figure 1:**
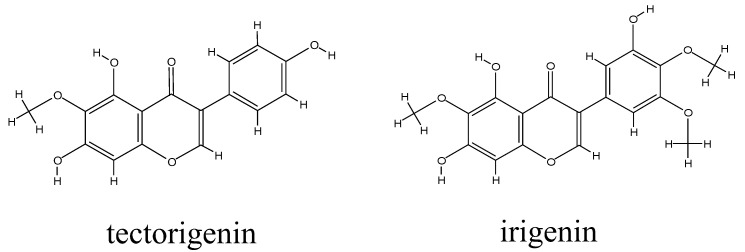
Structures of tectorigenin and irigenin.

To date, more than 100 compounds, including isoflavonoids, quinones, and terpenoids, have been identified in Shegan [2]. Isoflavonoids are considered the major active components, among which tectorigenin and irigenin are the key and most studied ingredients [2]. Tectorigenin (Figure 1) shows good anti-inflammatory [4], antioxidant [5], and antihyperlipidemic [6] activities and demonstrates protective effects on the lungs [7], thus contributing to the overall therapeutic power of Shegan [8]. Recent evidence suggests that tectorigenin exerts protective effects on the liver by regulating Toll-like receptor 4/NF-κB signaling and promoting autophagy [9], whereas, in the kidney, it inhibits Smad3-mediated ferroptosis and suppresses fibrosis [10]. Irigenin (Figure 1), another active isoflavonoid in Shegan, also shows anti-inflammatory activity by downregulating iNOS/COX-2 expression and NF-κB activation. Furthermore, irigenin alleviates doxorubicin-induced cardiotoxicity by inhibiting apoptosis and oxidative stress through upregulation of miR-425 [11] and suppresses the migration and invasion of lung cancer cells by modulating epithelial-to-mesenchymal transition [12]. These pharmacological studies indicate that tectorigenin and irigenin are potent therapeutic agents for various diseases.

Drug metabolism is an important part of pharmacokinetic research and is considered one of the key factors affecting the bioavailability of target compounds [13]. Understanding the metabolic pathways of drugs, including enzymes involved in their biotransformation and the resulting metabolites, is fundamental for predicting the therapeutic effect, screening candidate compounds, and revealing potential interactions between herbal components and other drugs. As the interest in tectorigenin and irigenin increases, it is particularly important to clarify their metabolism in the organism. Previous studies on tectorigenin pharmacokinetics in rats showed that the plasma levels of conjugated tectorigenin metabolites (glucuronide and sulfate) are much higher than that of tectorigenin aglycone [14], and that the concentration of tectorigenin monoglucuronide is higher than that of other metabolites [15]. On the other hand, no studies have investigated the metabolites of irigenin in vivo. It is worth mentioning that one report on iridin, the glucoside of irigenin, indicated that irigenin is also metabolized into glucuronide conjugates, and that some UDP-glucuronosyltransferase (UGT) enzymes are involved in its metabolism [16]. These reports indicate that glucuronidation may have a key role in the elimination of irigenin and tectorigenin in vivo. Nevertheless, the enzymes involved in the glucuronidation of tectorigenin and irigenin and their enzyme kinetic have not been investigated.

The aim of this study was to identify the enzymes involved the metabolism of tectorigenin and irigenin and their kinetic parameters. The results should promote further research in this area and contribute to the development of new drugs based on tectorigenin and irigenin.

## 2. Results

### 2.1. Metabolic Profiles of Tectorigenin and Irigenin

**Figure 2:**
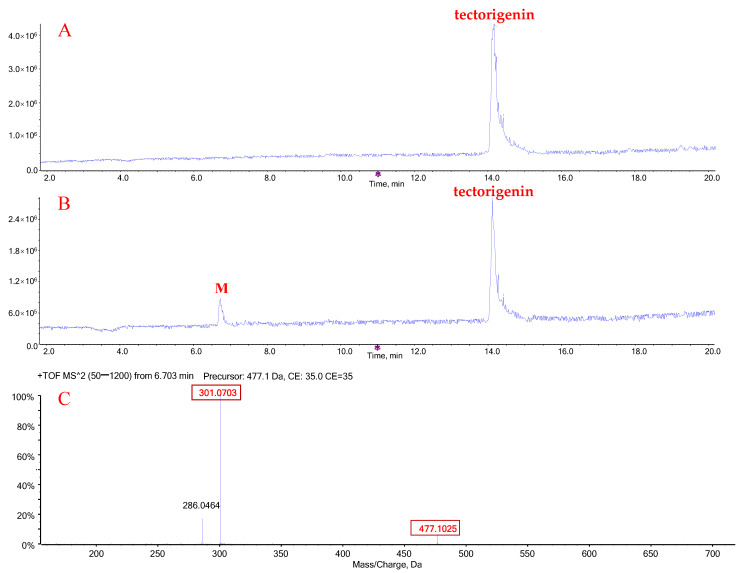
Representative chromatograms of tectorigenin and its glucuronide metabolites (M). Total ion chromatogram (TIC) after incubation of tectorigenin for 0 h (**A**) and 0.5 h (**B**) at 37 °C; mass spectrometric fragmentation characteristics of glucuronide metabolite of tectorigenin (**C**).

When tectorigenin was incubated with human liver microsomes (HLMs) in the presence of uridine diphosphoglucuronic acid (UDPGA), a new obvious peak (M) appeared at 6.7 min, which was absent in the control (no UDPGA) (Figure 2A,B). Mass spectrometry analysis in the positive-ion mode revealed that the accurate molecular weight of metabolite M was 477.1025 Da ([M + H]^+^), which was 176.0322 Da higher than that of tectorigenin, with a product ion at *m*/*z* 301.0703 (Figure 2C). These data suggest that the new metabolite may have resulted from tectorigenin glucuronidation.

**Figure 3:**
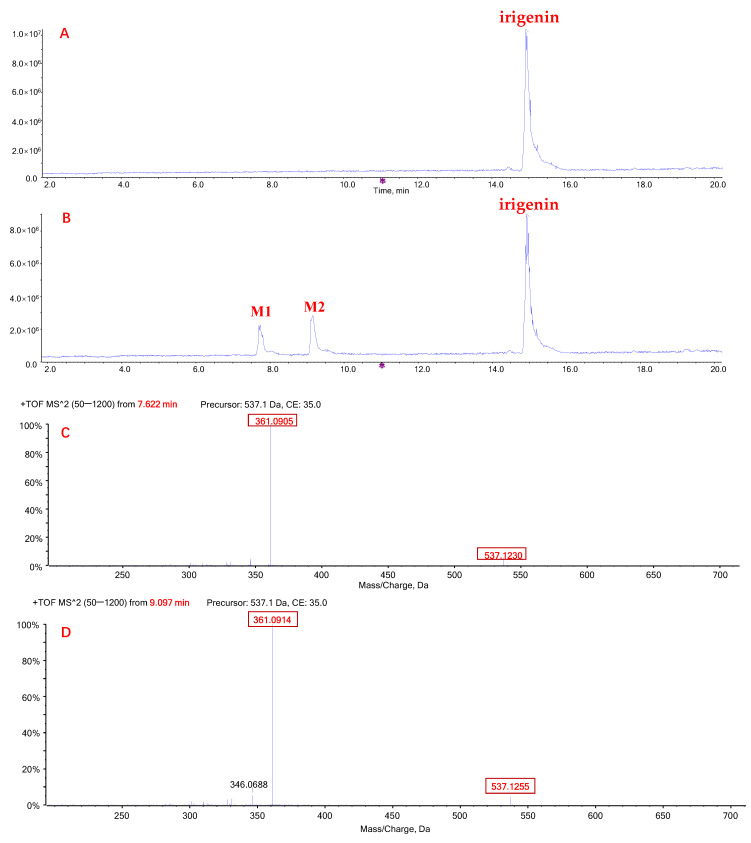
Representative chromatograms of irigenin and its glucuronide metabolites M1 and M2. Total ion chromatogram (TIC) after incubation of tectorigenin for 0 h (**A**) and 0.5 h (**B**) at 37 °C; mass spectrometric fragmentation characteristics of glucuronide metabolite M1 (**C**) and M2 (**D**) of tectorigenin.

When irigenin was incubated with HLMs and UDPGA, two metabolites (M1 and M2) were detected (Figure 3A,B). M1 (retention time: 7.7 min) and M2 (retention time: 9.1 min) had the same exact molecular weight, 537.12 Da ([M + H]^+^), which was 176.03 Da higher than that of irigenin. The most abundant product ions of M1 and M2 were both 361.09 Da and were generated by a neutral loss of 176 Da (Figure 3C,D), indicating that the two metabolites were formed by glucuronidation of irigenin.

### 2.2. Response Factor Values

As it was difficult to obtain sufficient amounts of tectorigenin and irigenin glucuronides for quantitative analysis, the response factor (RF) method was used to determine the concentration of the produced metabolites [17,18]. RF values were calculated on the basis of the ratio of the peak areas of tectorigenin and irigenin obtained after hydrolysis of their glucuronides to those of the glucuronides (drug to metabolite). The RF value for tectorigenin to glucuronide M was 0.92, while those for irigenin to glucuronides M1 and M2 were 0.87 and 0.90, respectively (Table 1).

### 2.3. Quantification of Metabolites

The glucuronide metabolites were quantified using the calibration curve of tectorigenin or irigenin, and the concentrations of these metabolites were corrected using the RF values. The quantification method was validated. The accuracies were represented by relative error (RE), and the precisions were represented by relative standard deviation (RSD). As a result, for both tectorigenin and irigenin, the lower quantification limit was 0.03 μM, and the calibration range was 0.03–10 μM. The precision represented by RSD was lower than 6.21% and 4.37% for tectorigenin and irigenin, respectively. The accuracies of tectorigenin and irigenin ranged from −3.70% to 2.13% and from −2.23% to 4.31%, respectively. The mean extraction recoveries of tectorigenin and irigenin were both greater than 97.3%.

In the stability experiment, we evaluated the stability of tectorigenin and irigenin, as well as their metabolites, under different conditions. The RE and RSD values for tectorigenin and irigenin in pretreated samples placed in the autosampler for 12 h at 4 °C were both below ±5%, which indicated that tectorigenin and irigenin were both stable. The stability results of metabolites of the experiment are presented in Table 2. Metabolites (M, M1, and M2) were stable in the inactivated metabolic system for 2 h (held constant at approximately 25 °C) and after storage for 7 days (−80 °C), as well as in the pretreated samples placed in the autosampler for 12 h at 4 °C.

### 2.4. Chemical Inhibition Studies

**Figure 4:**
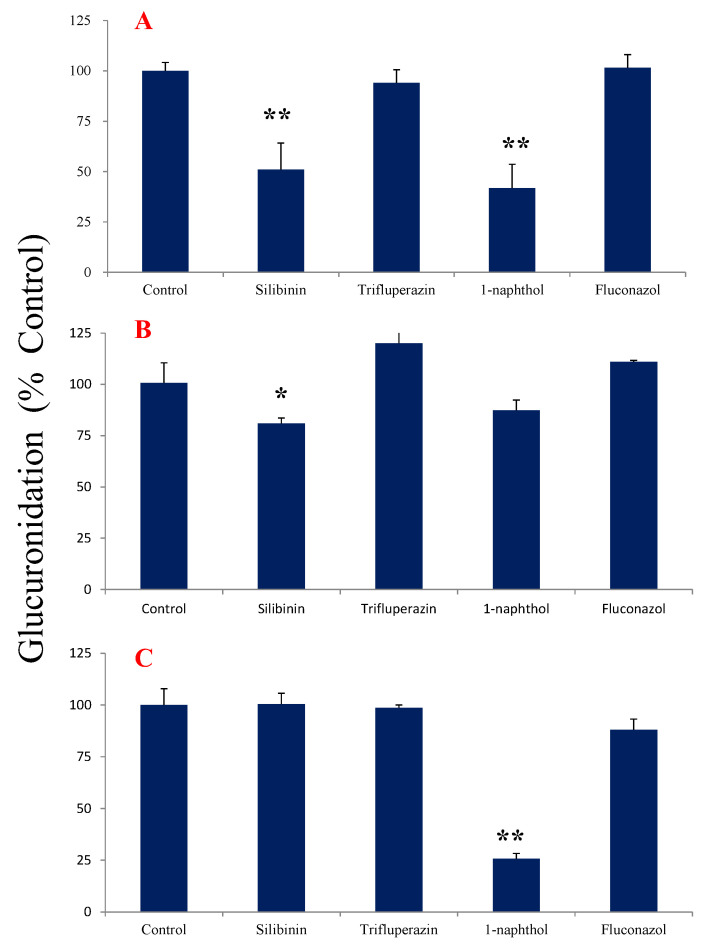
Effect of UGT inhibitors on tectorigenin and irigenin glucuronidation in HLMs (*n* = 3; data represent the mean ± SE). (**A**) Tectorigenin with and without inhibitor; (**B**) irigenin with and without inhibitor for metabolite 1 of irigenin; (**C**) irigenin with and without inhibitor for metabolite 2 of irigenin. * *p* < 0.05, ** *p* < 0.01 vs. control.

Figure 4A exhibited the effects of UGT inhibitors on the metabolic reaction of tectorigenin in HLMs. Silibinin (an inhibitor of UGT1A1) and 1-naphthol (an inhibitor of UGT1A6 and UGT1A9) significantly inhibited glucuronidation, indicating that UGT1A1 and UGT1A6 or UGT1A9 may participate in the glucuronidation of tectorigenin.

In case of irigenin, silibinin significantly inhibited the production of M1 (Figure 4B), whereas 1-naphthol inhibited that of M2 (Figure 4C), suggesting that the formation of M1 and M2 was mediated by different metabolic enzymes. The other inhibitors had no obvious effects on the glucuronidation of irigenin.

### 2.5. Glucuronidation by UGTs

**Figure 5:**
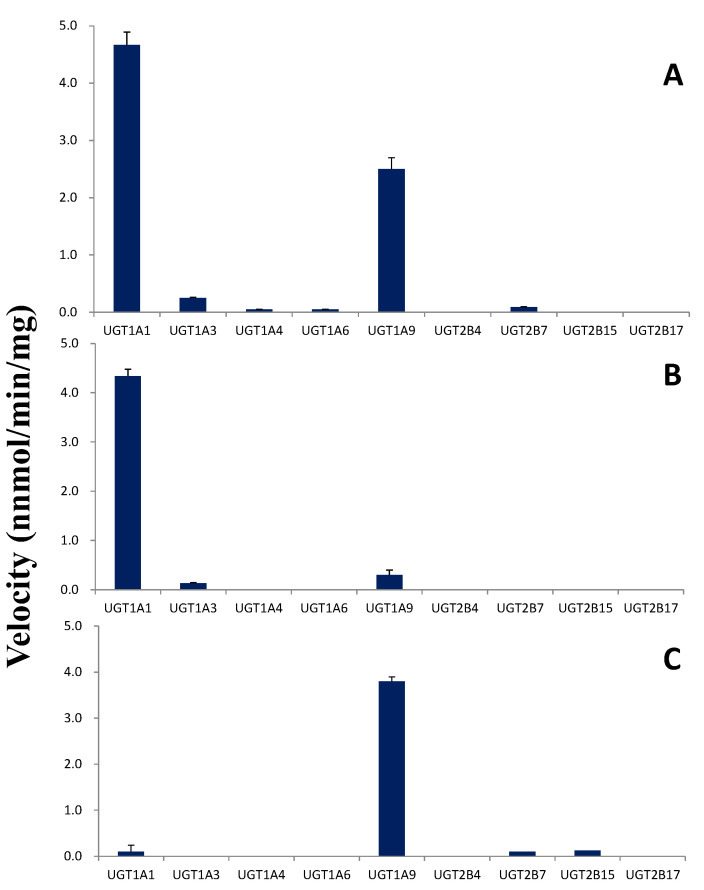
Screening of glucuronidation metabolism of tectorigenin and irigenin with eight recombinant enzymes (*n* = 3; data represent the mean ± SE). (**A**) Velocity for the formation of the metabolite of tectorigenin; (**B**) velocity for the formation of metabolite 1 of irigenin; (**C**) velocity for the formation of metabolite 2 of irigenin.

Next, UGTs were used to determine the subtypes participated in the metabolic reaction of tectorigenin and irigenin. In glucuronidation of tectorigenin, UGT1A1 and UGT1A9 had the highest activity, and UGT1A3, UGT1A4, UGT1A6, and UGT2B7 had some activity, whereas UGT2B4, UGT2B15, and UGT2B17 had none (Figure 5A). In glucuronidation of irigenin, the subtypes catalyzing M1 formation were UGT1A1, UGT1A3, and UGT1A9, among which UGT1A1 had the highest activity (Figure 5B), whereas the subtypes catalyzing M2 formation were UGT1A1, UGT1A9, UGT2B7, and UGT2B15, with UGT1A9 demonstrating the highest effect (Figure 5C).

### 2.6. Kinetic Studies in HLMs and UGTs

**Figure 6:**
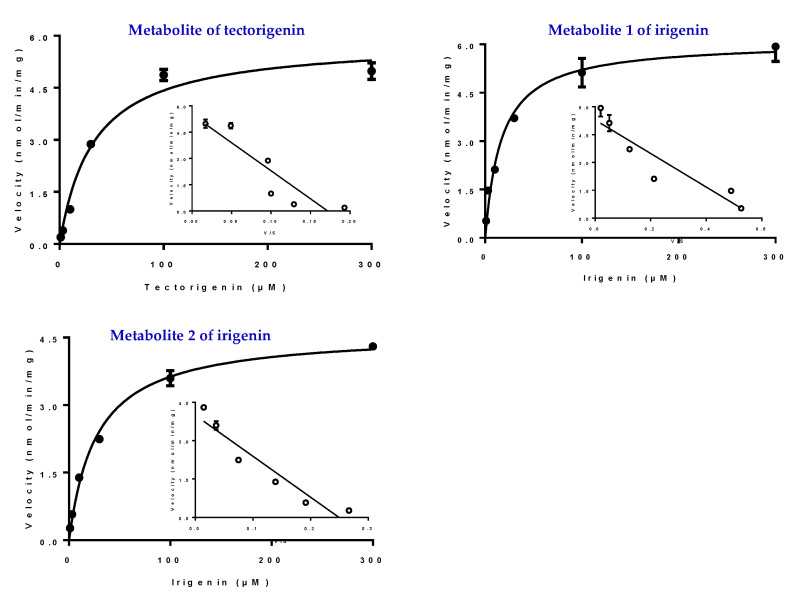
Kinetic profiles for formation of glucuronidation of tectorigenin and irigenin in HLMs (the filled circle). In each panel, the inset figure shows the corresponding Eadie–Hofstee plot (the hollow circle). All experiments were performed in triplicate. Data represent the mean ± SE (*n* = 3).

Kinetic studies on the glucuronidation metabolites of tectorigenin and irigenin were conducted using HLMs. Kinetic profiling revealed that the glucuronidation of tectorigenin and irigenin was best fitted using the Michaelis–Menten equation. The results of an Eadie–Hofstee plot supported the Michaelis–Menten kinetic characteristics (Figure 6). The enzyme kinetic parameters Michaelis–Menten constant (*K_m_*), maximum velocity (*V_max_*), and intrinsic clearance (*CL_int_* = *V_max_*/*K_m_*) for M formation were 32.79 ± 4.79 μM, 5.87 ± 0.25 nmol/min/mg, and 180 μL/min/mg, respectively, whereas those for M1 and M2 formation were 16.86 ± 2.96 μM, 6.09 ± 0.27 nmol/min/mg, and 360 μL/min/mg, and 27.37 ± 2.46 μM, 1.16 ± 0.03 nmol/min/mg, and 420 μL/min/mg, respectively.

**Figure 7:**
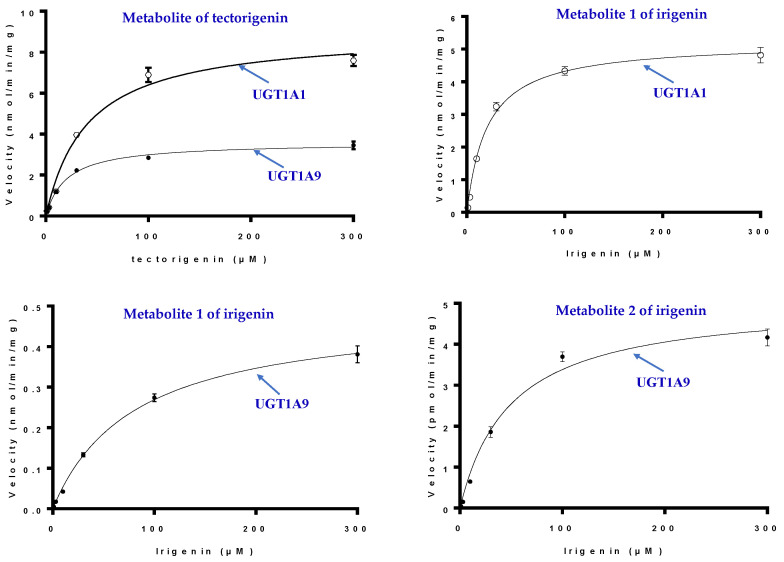
Kinetics of tectorigenin and irigenin metabolism by UGT1A1 and UGT1A9 (mean ± SE, *n* = 3) (the hollow circle for UGT1A1 and the filled circle for UGT1A9).

The detailed kinetics parameters of UGT1A1 and 1A9 for M, M1, and M2 are shown in Table 3 and Figure 7. The *K_m_* and *V_max_* values for tectorigenin glucuronidation with UGT1A1 and UGT1A9 were higher and lower, respectively, than those with HLMs, indicating that UGT1A1 and UGT1A9 co-mediated tectorigenin metabolization. The results also revealed that UGT1A1 had better affinity to irigenin and higher catalytic activity in producing M1 than UGT1A9, suggesting that M1 formation was mainly mediated by UGT1A1. In addition, the enzyme activity of UGT1A9 in producing M2 was similar to that of HLMs.

## 3. Discussion

Metabolic biotransformation is the main pathway of drug elimination from the body and can be subdivided into phase I and phase II. Phase I metabolism is mainly mediated by cytochrome P450 systems and comprises functional group reactions, including oxidation–reduction and hydrolysis [19]. Phase II metabolism consists of conjugation reactions, when drugs are covalently bound to endogenous molecules to form highly polar metabolites that could be easily eliminated from the body because of their hydrophilic nature [20]. Approximately 40–70% of the drugs entering the organism are cleared in phase II metabolism through the activity of various enzymes, among which UGTs catalyzing glucuronidation are the most important, being responsible for approximately 35% of all phase II reactions [21,22]. A previous study found that UGT-mediated conjugation reaction is one of the main pathways in flavonoid metabolism [23].

Tectorigenin (5,7,4′-trihydroxyl-6-methoxyl isoflavone) [24] and irigenin (3’,5,7-trihydroxy-4’,5’,6-trimethoxy isoflavone) are the key isoflavonoids in Shegan. In terms of chemical structure, both tectorigenin and irigenin contain phenolic hydroxyl groups, which are very prone to glucuronidation. Previous studies have shown that tectorigenin undergoes extensive phase II metabolism, and that its monoglucuronide conjugate is the major metabolite [14,15]. Irigenin can also be metabolized by UGTs [16]. However, there have been no studies on the kinetic parameters of enzymes involved in the metabolization of tectorigenin and irigenin in humans. Therefore, in this work, we analyzed the kinetics of tectorigenin and irigenin glucuronidation by HLMs and recombinant UGTs.

After the coincubation of tectorigenin with HLMs, one major glucuronidation metabolite was confirmed and detected by high-performance liquid chromatography coupled with TripleTOF 5600 quadrupole-time-of-flight mass spectrometry (Q-TOF-MS) and quantified using the RF method. RF values were stable, which demonstrated that using a prototype drug with its respective RF is a valid means to accurately calculate the parameters of tectorigenin. Chemical inhibition and recombinant enzyme experiments showed that multiple UGT subtypes exhibited glucuronidation activity toward tectorigenin; among them, UGT1A1 and UGT1A9 had the strongest catalytic capacity. Since both UGT1A1 and UGT1A9 are highly expressed in the liver [25] (6.7% and 6.1% of the total expression of UGTs, respectively), they might be the main subtypes involved in tectorigenin metabolism. As standards to determine the metabolite structure were lacking, we made assumptions according to the literature. A previous study indicated that glucuronidation is a key reaction in tectorigenin metabolism, and the concentrations of tectorigenin conjugates in plasma are higher than that of tectorigenin [14,15]. Because tectorigenin-7-*O*-glucuronide and tectorigenin-7-*O*-sulfate are the primary metabolites, the C-7 position might be the main site targeted by glucuronidation, even though there are three phenolic hydroxyl groups available. Therefore, we speculated that, in the present study, M was tectorigenin-7-*O*-glucuronide.

When irigenin was incubated with HLMs, two major metabolites (M1 and M2) were detected. These results are consistent with a previous in vivo study showing that irigenin, the deglycosylation product of iridin, is metabolized into two glucuronide conjugates [16]. Similar to tectorigenin glucuronide, the concentrations of M1 and M2 were quantified using the RF method. Although both M1 and M2 were the products of irigenin glucuronidation, the effects of the same inhibitors were different, suggesting that distinct sites were targeted by distinct enzymes. This notion was confirmed in the experiments with UGT subtypes, indicating that M1 was mostly produced by UGT1A1, whereas M2 was mostly produced by UGT1A9. Although a previous study showed that the glucuronidation of irigenin is also catalyzed by UGT1A7, UGT1A8, and UGT1A10 [16], the expression of these enzymes in the human liver is low [26]. Thus, it can be suggested that UGT1A1 and UGT1A9 are the major drug-metabolizing enzymes responsible for the formation of irigenin-*O*-glucuronide.

Tectorigenin and irigenin show good pharmacological activity and have been identified as candidate bioactive compounds [27]; however, there are few reports on the metabolism of these compounds. The results of our study help clarify the metabolization of tectorigenin and irigenin, which is crucial for the prevention of drug–drug interactions and the development and safe use of these isoflavonoids. In general, glucuronidation is considered a mechanism of detoxification and inactivation of exogenous compounds. However, there are exceptions such as morphine-6-glucuronide, which is more pharmacologically active than the original morphine [28]. Therefore, the functional activities of tectorigenin and irigenin metabolites await further investigation.

## 4. Materials and Methods

### 4.1. Materials

Tectorigenin and irigenin were supplied by Yuanye Biotechnology Co., Ltd. (Shanghai, China). UDPGA, silibinin, 1-naphtol, fluconazole, trifluoperazine, and β-glycosidase were obtained from Sigma-Aldrich (St. Louis, MO, USA). Alamethicin and d-glucaric acid 1,4-lactone monohydrate were purchased from Meilunbio (Shanghai, China). Pooled HLMs (mixed gender, protein concentration: 15 mg/mL) were purchased from the Research Institute for Liver Diseases (Shanghai, China). Nine recombinant human UGT enzymes (UGT1A1/1A3/1A4/1A6/1A9/2B4/2B7/2B15/2B17) were obtained from BD Biosciences (Woburn, MA, USA). HLMs and recombinant UGTs were stored in a freezer (−80 °C) until use. All other reagents were supplied by the Sinopharm Group (Shanghai, China).

### 4.2. Metabolic Stability Experiments

To analyze metabolic stability, tectorigenin and irigenin were incubated with HLMs to explore their metabolic stability, as previously described [18,29]. In short, tectorigenin or irigenin (100 μM) was incubated in 50 mM Tris-HCl buffer (pH 7.4) containing MgCl_2_ (10 mM), HLMs (0.5 mg/mL), alamethicin (50 μg/g protein), 1,4-lactone monohydrate (5 mM), and UDPGA (1 mM). After the mixture was incubated for 30 min (37 °C), the reaction was stopped by ice-cold methanol. After centrifugation, the supernatant was used to qualitatively analyze the metabolites.

### 4.3. Molecular Weight Analysis of Tectorigenin and Irigenin Glucuronides

The molecular weight of tectorigenin and irigenin glucuronides was determined using the HPLC–Q-TOF-MS method. Chromatographic analysis was conducted using an ODS C_18_ column (150 × 4.6 mm, 5 μm); mobile phase A was 0.1% acetic acid and 2 mM ammonium acetate in water, while mobile phase B was acetonitrile. Gradient elution was performed as follows: 0–5 min, 90% to 80% A; 5–20 min, 80% to 50% A; 20–21 min, 50% to 40% A; 21–25 min, 40% to 10% A. Q-TOF-MS in positive electrospray ionization source (ESI mode) was used with a generic method for data acquisition. The mass range from *m*/*z* 100 to *m*/*z* 1200 was fully scanned. Data were acquired and processed using Analyst and Peakview (AB Sciex), respectively. 

### 4.4. Determination of RF Values

As it is difficult to obtain sufficient amounts of tectorigenin and irigenin glucuronides for quantitative analysis, the RF method was used to calculate their concentrations. Tectorigenin and irigenin glucuronides were prepared with HLMs (1.0 mg/mL) in the reaction described in Section 4.2. The two concentrations of tectorigenin and irigenin were 30 μM and 300 μM. After 90 min, the enzymatic reaction was stopped, the supernatant was analyzed, and metabolite fractions were collected automatically as previously described [18].

A part of each fraction (200 μL) was co-incubated with β-glucuronidase (1000 U/mL) for 12 h at 37 °C to hydrolyze the metabolites and determine the peak areas of tectorigenin or irigenin (A_prototype drug_), whereas the remainder was directly injected into the column to determine the peak areas of the metabolite (A_metabolite_). The RF was expected be similar at different concentrations, and was calculated as follows:

RF = A_prototype drug_/A_metabolites_, (1)

where A_metabolites_ and A_prototype drug_ are the peak areas of metabolites and prototype drugs (tectorigenin and irigenin), respectively.

### 4.5. Quantitative Determination of Metabolites of Tectorigenin and Irigenin

An HPLC method was used for the determination of metabolites of tectorigenin and irigenin. The settings of the chromatographic column and mobile phase were as described in Section 4.3. The detection wavelength was 260 nm.

Glucuronide metabolite was quantified on the basis of the calibration curve of tectorigenin or irigenin, and the concentrations of these metabolites were calculated as follows:

C_metabolites_ = [A _metabolites_ × RF − a]/b, (2)

where a and b are the intercept and slope, respectively, of the calibration curve of tectorigenin or irigenin, respectively, and A_metabolites_ is the peak area of the metabolites.

### 4.6. Glucuronidation Kinetics of Tectorigenin and Irigenin in HLMs

To obtain suitable incubation conditions, we first evaluated the linearity of metabolite formation at different times (10–60 min), the UDPGA concentrations (0.5–5 mM), and the microsome protein concentrations in HLMs (0.2–2 mg/mL). The final incubation medium used in the experiment contained MgCl_2_ (10 mM), microsomes (0.5 mg/mL), 1,4-lactone monohydrate (5 mM), alamethicin (50 μg/g protein), UDPGA (1 mM), and different concentrations of tectorigenin or irigenin (1–300 μM) in Tris-HCl buffer (50 mM, pH 7.4). The mixture was incubated at 37 °C for 30 min; then, the reaction was stopped by adding ice-cold methanol. After the mixtures were centrifuged (17,000 rpm, 10 min), and the supernatant was analyzed by HPLC. Michaelis–Menten parameters (*V_max_* and *K_m_*) were determined on the basis of the nonlinear regression of the plot of the substrate concentration vs. rate of metabolite formation.

### 4.7. Inhibition of Glucuronidation Reaction by Chemical Inhibitors

Several well-characterized UGT substrates were used as “inhibitors” to examine the involvement of UGTs [30] in the glucuronidation of tectorigenin or irigenin. To reduce nonspecific inhibition, the concentration of each inhibitor was determined according to the available literature, namely, 100 μM silibinin for UGT1A1 [31], 50 μM trifluoperazine for UGT1A4 [32], 100 μM 1-naphthol for UGT1A6/1A9 [18,33], and 500 μM fluconazole for UGT2B7 [34]. A reaction without inhibitors was carried out in parallel as a control.

### 4.8. UGT Reaction Screening and Enzyme Kinetics of Tectorigenin and Irigenin

UGT reaction screening of tectorigenin and irigenin was conducted using nine commercially available human UGTs (UGT1A1/1A3/1A4/1A6/1A9/2B4/2B7/2B15/2B17). Furthermore, we studied the enzymatic kinetic characteristics of tectorigenin and irigenin using UGT1A1 and UGT1A9. Six concentrations of tectorigenin and irigenin (1, 3, 10, 30, 100, and 300 μM) were incubated in triplicate in order to obtain the enzyme kinetic characteristics of tectorigenin and irigenin glucuronide.

### 4.9. Data Analysis

For both HLMs and UGTs, metabolic kinetic analysis was performed by fitting the data to the Michaelis-Menten model. The *Km* and *Vmax* of enzymes were calculated as follows:

(3) $$V=V\max\times \frac{\left[S\right]}{Km+[S]},$$

where [S] is the substrate concentration. All results were shown as the mean ± standard error (SE). Differences among different groups were compared using one-way analysis of variance. A *p*-value < 0.05 was considered statistically significant.

## 5. Conclusions

This study provides important information on the glucuronidation profiles of tectorigenin and irigenin, two important isoflavones in Shegan. In HLMs, tectorigenin and irigenin were converted to one and two glucuronides, respectively. UGT1A1 and UGT1A9 were identified as the main enzymes catalyzing glucuronidation of tectorigenin and irigenin. Both enzymes could produce tectorigenin glucuronide M, whereas UGT1A1 mostly generated irigenin glucuronide M1, while UGT1A9 mostly generated irigenin glucuronide M2. These results point to the need of monitoring drug pharmacokinetics when a Chinese medicine containing these two isoflavones is co-administered with clinical drugs sensitive to UGT1A1- and/or UGT1A9-mediated glucuronidation.

## Figures and Tables

**Table 1:** Response factors of glucuronidation metabolites to tectorigenin and irigenin (*n* = 3).

Response Factors		Mean ± SE	Mean
A_tectorigenin_/A_M_	Low	0.91 ± 0.02	0.92
	High	0.92 ± 0.02	
A_irigenin_/A_M1_	Low	0.87± 0.04	0.87
	High	0.87 ± 0.06	
A_irigenin_/A_M2_	Low	0.88 ± 0.04	0.90
	High	0.91 ± 0.06	

A_tectorigenin_ and A_irigenin_ are the peak areas of tectorigenin and irigenin, respectively. A_M_, A_M1_, and A_M2_ are the peak areas of the glucuronidation metabolite of tectorigenin, glucuronidation metabolite 1 of irigenin, and glucuronidation metabolite 2 of irigenin, respectively.

**Table 2:** The stability of metabolites under different storage conditions (*n* = 5).

Compounds	Concentration	in Inactivated Metabolic System for 2 h		−80 °C for 7 Days		in Autosampler for 12 h	
		RSD (%)	RE (%)	RSD (%)	RE (%)	RSD (%)	RE (%)
M	Low	2.13	0.38	3.02	2.90	5.27	−2.13
	High	2.08	0.59	1.97	4.71	1.08	−5.18
M1	Low	3.21	−3.43	2.86	−2.01	3.41	−2.40
	High	1.09	2.10	6.23	1.84	2.15	−3.06
M2	Low	4.32	1.89	3.04	−2.85	5.57	−2.98
	High	1.92	−2.07	1.95	−3.49	3.29	−5.07

M, M1, and M2 are the glucuronidation metabolite of tectorigenin, glucuronidation metabolite 1 of irigenin, and glucuronidation metabolite 2 of irigenin, respectively.

**Table 3:** Enzyme kinetic parameters of tectorigenin and irigenin glucuronidation (mean ± SE, *n* = 3).

Substrate	Enzyme	*K_m_*	*V_max_*
		(μM)	(nmol/min/mg Protein)
Tectorigenin	UGT1A1	40.64 ± 5.56	9.01 ± 0.39
	UGT1A9	18.37 ± 1.16	3.49 ± 0.58
Irigenin	UGT1A1(M1)	20.86 ± 2.04	5.22 ± 0.14
	UGT1A9(M1)	73.88 ± 4.04	0.45 ± 0.09
	UGT1A9(M2)	25.04 ± 2.37	4.56 ± 0.12

## Data Availability

Data are available from the authors.

## Sample Availability

Samples of the compounds are available from the authors.
